# Golgin-97 Targets Ectopically Expressed Inward Rectifying Potassium Channel, Kir2.1, to the *trans*-Golgi Network in COS-7 Cells

**DOI:** 10.3389/fphys.2018.01070

**Published:** 2018-08-03

**Authors:** Tarvinder K. Taneja, Donghui Ma, Bo Y. Kim, Paul A. Welling

**Affiliations:** Department of Physiology, Maryland Center for Kidney Discovery, School of Medicine, University of Maryland, Baltimore, Baltimore, MD, United States

**Keywords:** potassium channel, golgi apparatus, clathrin, inward rectifying K channel, membrane trafficking

## Abstract

The inward rectifying potassium channel, Kir2.1, is selected as cargo at the *trans*-Golgi network (TGN) for export to the cell surface through a unique signal-dependent interaction with the AP1 clathrin-adaptor, but it is unknown how the channel is targeted at earlier stages in the secretory pathway for traffic to the TGN. Here we explore a mechanism. A systematic screen of Golgi tethers identified Golgin-97 as a Kir2.1 binding partner. *In vitro* protein-interaction studies revealed the interaction is direct, occurring between the GRIP domain of Golgin-97 and the cytoplasmic domain of Kir2.1. Imaging and interaction studies in COS-7 cells suggest that Golgi-97 binds to the channel en route through the Golgi. RNA interference-mediated knockdown of Golgin-97 prevented exit of Kir2.1 from the Golgi. These observations identify Golgin-97 as a Kir2.1 binding partner that is required for targeting the channel to the TGN. Based on our studies in COS-7 cells, we propose Golgi-97 facilitates formation of AP1-dependent export carriers for Kir2.1 by coupling anterograde delivery of Kir2.1 with retrograde recycling of AP-1 containing endosomes to the TGN.

## Introduction

Highly orchestrated trafficking processes ensure that cardiac ion channels are expressed on appropriate membrane domains at suitable densities to maintain the electrical rhythm of the heart. Targeting of the inward rectifying potassium channel, Kir2.1, to the t-tubule and sarcolemma in ventricular myocytes (Ma et al., 2011), for example, is made possible by a series of sequential processes that deliver newly synthesized channels from the endoplasmic reticulum to the Golgi (Ma et al., 2001), control *trans*-Golgi network (TGN) to the cell surface (Ma et al., 2011), and drive association with appropriate lipid domains (Vaidyanathan et al., 2013, 2018; Han et al., 2014) and scaffolding complexes (Leonoudakis et al., 2004a,b; Rubi et al., 2017). The importance of membrane trafficking is underscored by human disease; mutations in Kir2.1 that disrupt proper trafficking (Bendahhou et al., 2003; Ballester et al., 2006; Ma et al., 2011) cause Andersen–Tawil syndrome (Plaster et al., 2001), which predisposes affected patients to cardiac arrhythmias.

Although great progress has been made in unraveling many of the mechanisms that control the transport of Kir2.1 in the secretory pathway, it still remains poorly understood how the channel is sorted in the Golgi from resident Golgi-proteins for transport to the TGN. Differences in transmembrane spans and lipid partitioning (Patterson et al., 2008) are generally believed to provide a universal mechanism. In the present study, we consider a new Golgi segregation process, involving Golgin molecules. Members of the Golgin protein family are well appreciated to act as specific tethers that selectively capture transport vesicles for SNARE-mediated fusion with distinct membrane compartments of the Golgi (Munro, 2011; Witkos and Lowe, 2015; Gillingham and Munro, 2016). Different Golgins associate with different Golgi regions and have distinct capture activities toward vesicles of different origins (Wong and Munro, 2014). Additionally, anterograde trafficking of several proteins through the Golgi, including the HERG cardiac potassium channel (Bundis et al., 2006), require specific Golgin molecules.

In the course of defining the TGN export mechanism in Kir2.1 (Ma et al., 2011) and related channels (Li et al., 2016), we made a serendipitous observation that implicated Golgin-97 in intra Golgi trafficking. We found mutations in the TGN export signal caused marked accumulation of the channel throughout the entire Golgi apparatus, suggesting export from the TGN might be closely coupled to anterograde transport through the Golgi. Remarkably, the Golgi-export mutants in the *cis*-Golgi tightly co-localized with Golgi-97, a golgin molecule that is usually confined to the TGN, making us wonder if a Golgin-97 interaction mechanism might explain how channels are selected in the Golgi for delivery to the TGN. Here, we test this idea.

## Materials and Methods

### Antibodies

Antibodies used in this study were: anti-HA mouse monoclonal antibody (Covance Inc., Princeton, NJ, United States), anti-HA rabbit monoclonal (Upstate Cell Signaling Solutions, Waltham, MA, United States), anti-myc rabbit polyclonal, (Santa Cruz Biotechnology, Inc., Santa Cruz, CA, United States), anti-Golgin-97 (Invitrogen, #A21270), anti-p230 (BD Transduction Laboratories, Lexington, KY, United States #611230), anti-GM130 (BD Transduction Laboratories, #51-9001978), horseradish peroxidase (HRP)-conjugated goat anti-mouse or anti-rabbit (Jackson Laboratory, Bar Harbor, ME, United States), Goat anti-GST-HRP (Amersham Biosciences, Piscataway, NJ, United States), Alexa Fluor-conjugated secondary antibodies (Invitrogen Molecular Probes, Eugene, OR, United States).

### Molecular Biology

All Kir2.1 constructs are derived from the mouse Kir2.1 channel cDNA (NM_008425). As described before (Ma et al., 2001), an external hemagglutinin (HA) epitope tag was engineered into the channel at a location that does not affect that the physiological function or biochemical properties of the channel (Ma et al., 2007). For some interaction studies, a myc-tag was incorporated in frame on to the N-terminus of Kir2.1 cytoplasmic C-terminal domain fragments. cDNAs for human Golgin-97 (MGC 22154), ARL1 (MGC 12286), and Golgi Matrix Protein 130 (Golga2, MGC 78452) were acquired from distributors of the IMAGE collection, and mouse p230/Golgin 245 was purchased from ATCC (10089301), and tagged in frame to the ORFs at the N or C-termini with HA or myc-epitopes. Site-directed mutagenesis was carried out using a PCR based strategy with PfuTurbo DNA polymerase (QuikChange, Stratagene). The pcDNA3.1 mammalian expression vector (Invitrogene, Inc.) was used to express the cloned channel genes in COS7 cells. For GST pull-down analyses, recombinant DNA fragments were subcloned into pGEX-5X-2. All sequences were confirmed by dye termination DNA sequencing analysis (University of Maryland School of Medicine Biopolymer Core).

### Cell Culture and Transfection

COS7 cells were cultured in high glucose DMEM medium (Invitrogene, Inc., Carlsbad, CA, United States) supplemented with 10% Fetal Bovine Serum, 100 U/ml penicillin and 100 μg/ml streptomycin in a humidified atmosphere at 37°C in 5% CO2. Cells were transfected using Fugene 6 or X-tremeGENE 9 transfection reagent (Roche Ltd., Basel, Switzerland).

### RNA Interference

The double-stranded siRNA to target human form of Golgin-97 and non-targeting siRNA were purchased from Thermo/Dharmacon Inc. (Lafayette, CO, United States). The Golgi-97 probe corresponded to sense sequence 5′-GAUCACAGCCCUGGAACAAUU-3′. Cells were transfected with ∼160 pmole of either probe, and transfected on the subsequent day with Kir2.1 and/or non-targeting Golgin cDNAs, and then assayed after an additional ∼36 h.

### Immunoprecipitation

COS-7 cells were lysed in ice-cold detergent buffer (1% Triton X-100, 1× PBS, with protease inhibitors) and the clarified supernatant (15,000 g spin, 15 min) was incubated with Protein A + G beads (Cal Biochem, cat IP05), and 1 μg of immunoprecipitating antibody for 4 h rotating at 4°C. Beads were washed for 5 min with 1× PBS for three times; bound proteins were eluted with 2× Laemmli sample buffer for 30 min at room temperature; and the entire immunoprecipitate was run on a SDS-gel, and transferred to nitrocellulose for immunoblot analysis.

### Quantitative Chemiluminescence Detection of Surface Proteins

To quantify cell surface expression of Kir2.1 in mammalian cells, 36 h after transfection COS7 were fixed with 3% paraformaldehyde (PFA) for 15 min on ice, blocked on ice with blocking buffer (5% Fetal Bovine Serum in 1× PBS, 30 min), incubated with mouse monoclonal anti-HA (Covance Inc.; 1:300 dilution in blocking buffer, 1 h at room temperature), washed (with 1× PBS, three times for 5 min), incubated with goat anti-mouse IgG HRP-conjugated secondary antibody (1:1000 dilution, Jackson) (in blocking buffer, 30 min), and then extensively washed (1× PBS, 5 min/time for four times in total). After washing, the cells were scraped off from the plates and resuspended well into 500 μl 1× PBS. From the cell suspension, 10 μl of cell suspension was withdrawn and incubated with 100 μl of mixed SuperSignal ELISA Pico solution (Pierce Biotechnology, Inc., Rockford, IL, United States), and subsequently chemiluminescence signal was measured as previously described (Ma et al., 2007). To normalize the data for total protein expression level, western-blot analyses were carried out in parallel with anti-HA, or anti-Kir2.1 (Chemicon Inc.), and then quantified by densitometry analysis. Reported values are the average of triplicate transfections from three different experiments.

### Production of Recombinant GST Fusion Proteins and Pull-Down Analysis

The GST fusion proteins were purified on glutathione-agarose beads according to the standard protocol supplied from Amersham Biosciences, except using two times more lysis buffer than the original volume suggested in the protocol to maximize the yield. To purify the recombinant protein to high homogeneity, the GST beads were further washed with high salt buffer (0.5 M NaCl, 25 mM HEPES, and 2.5 mM MgCl_2_, pH7.0) twice before balancing with 1× KOAc buffer (115 mM KOAc, 25 mM HEPES, and 2.5 mM MgCl_2_, pH7.0) for pull-down analysis. The relative amount of fusion protein was quantitated by SDS-PAGE electrophoresis and then stained with coomassie blue in parallel with serial diluted BSA standard. For pull-down assays, 10 μg of purified GST beads were incubated with 100 μg of cellular lysates. After overnight incubation at 4°C, the GST beads were extensively washed with 1× PBS buffer containing 0.1% Triton X-100 to remove any non-specific binding (4 times for 10 min each time). Proteins bound on the beads were eluted by SDS-loading dye, fractionated by SDS-PAGE gel electrophoresis, blotted onto nitrocellulose membrane, and then probed with the relevant antibody.

## Results

### Kir2.1 Interacts Specifically With Golgin-97

**FIGURE 1:**
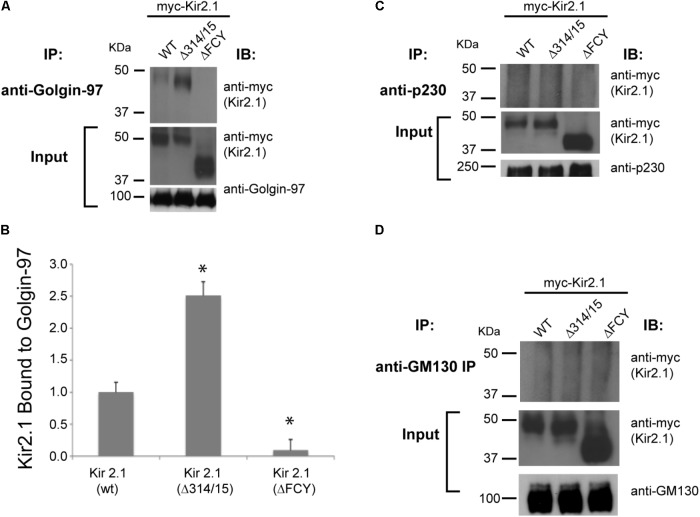
Kir2.1 specifically binds to Golgin-97. Immunoprecipitation (IP) performed with **(A)** anti-Golgin-97; **(C)** anti-p230; **(D)** anti-GM130 antibodies from COS cells transfected with myc-Kir2.1 wild type (WT), the TGN-export mutant (Δ314–315), and the ER-export mutant (ΔFCY) and then immunoblotted (IB) with myc-antibodies to detect Kir2.1 in the Golgin immunoprecipitates. 5% input is shown below; **(B)** Quantification of the relative amounts of Kir2.1 immunoprecipatated with Golgin-97. ^∗^*P* < 0.05.

We considered the possibility that Kir2.1 might be pre-selected as secretory cargo in the Golgi for export at the TGN by interaction with specific Golgin tethering molecules. To explore this hypothesis, we first surveyed members of the two main classes of Golgi tethers (GM130 and the GRIP domain containing Golgins, Golgin-245/p230, and Golgin-97) for interaction with Kir2.1. As measured by co-immunoprecipitation from COS7 cells that were transfected with Kir2.1, we found that the wild-type channel Kir2.1 (WT) and the TGN-export mutant (Δ314–315) channel but not the ER-export mutant (ΔFCY) interact with Golgin-97, as expected for an interaction that is initiated within the Golgi (**Figure 1A**). Consistent with this idea, we found more of the TGN-export mutant (Δ314–15) channel co-immunoprecipitated with Golgin-97 than the WT channel (**Figure 1B**). By contrast, we were unable co-immunoprecipitate Kir2.1 with antibodies to Golgin-245/p230 (**Figure 1C**), another TGN-localized, GRIP-containing Golgin (Cowan et al., 2002), or GM130 (**Figure 1D**), a member of the Golgin family of coiled-coil proteins that controls membrane tethering between the *cis*-Golgi intermediate compartment and endoplasmic reticulum and that has been implicated in HERG-channel trafficking (Newton-Cheh et al., 2009). Thus, Kir2.1/Golgin-97 interaction appears to be specific, at least amongst the Golgin molecules that were studied.

### Golgin-97 GRIP Domain Associates With the Cytoplasmic Domain of Kir2.1

**FIGURE 2:**
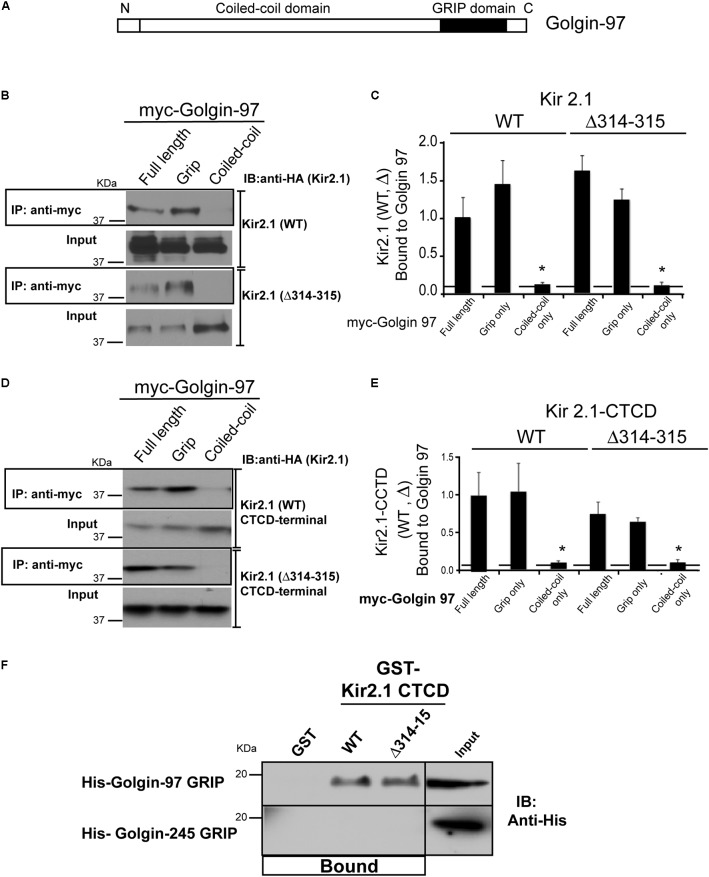
Golgin-97 GRIP domain directly interacts with Kir2.1. **(A)** Domain structure of Golgin-97, showing the GRIP domain and the coiled-coil domain. **(B)** Anti-myc immunoprecipitation (IP) followed by anti-HA immunoblotting (IB) from COS cells co-transfected with myc-Golgi 97 (Full length, Grip domain only, or coiled-coiled domain only) and HA-Kir2.1 wild type, WT, or the TGN-export mutant (Δ314–315) or **(D)** the HA-tagged Kir2.1 COOH-terminal cytoplasmic domain (CTCD). **(C,E)** Quantitative summaries comparing relative amounts of Kir2.1 (WT or Δ314–315) immunoprecipitated with full-length Golgin-97, or GRIP or Coiled-Coil domain, ^∗^*P* < 0.05. **(F)** GST pulldowns: GST alone or GST fusion proteins of the Kir2.1 CTCD were used to test for direct interaction with His-tagged GRIP domains of Golgin-97 or Golgin-245 purified from bacteria. Protein bound is shown relative to 5% input.

We next sought to determine the protein binding domains that govern the interaction between Kir2.1 and Golgi-97. Golgin-97 contains two major domains, the ∼45 residue GRIP domain, which binds to Arl1 to drive Golgi localization (Lu and Hong, 2003; Panic et al., 2003; Derby et al., 2004; Lu et al., 2004; Wu et al., 2004), and a long coiled domain, which provides a tethering function (Wong and Munro, 2014; **Figure 2A**). To test which Golgin-97 domain(s) interact with Kir2.1, COS7 were co-transfected different myc-tagged Golgin-97 constructs and HA-tagged Kir2.1, and anti-myc immunoprecipitations were followed by anti-HA immunoblotting. As shown in **Figure 2** (**Figure 2B** representative gel, **Figure 2C**, summary data, *n* = 3), the isolated GRIP domain of Golgin-97 but not the coiled-coil domain is sufficient for binding to Kir2.1. Both WT Kir2.1 and the TGN-export mutant (Kir2.1 Δ314–315) are able to interact with the GRIP domain.

We considered that the large C-terminal cytoplasmic domain (CTCD) to be the most likely structure in the channel to interact with Golgin-97, and, therefore tested if it was sufficient to interact with Golgin-97. For these studies, the HA-tagged C-terminal domain (WT or the Δ314–315 mutant) was co-expressed in COS7 cells with myc-tagged Golgin-97 (full length or isolated) and myc-antibody immunoprecipitations were performed (**Figure 2D**, summarized in **Figure 2E**). As detected in anti-HA Western blots of the myc-antibody immunoprecipitates, we found the Kir2.1 CTCD is able to interact with full length Golgin-97 through the GRIP domain. The Δ314–315 mutant, which lacks part of the Golgi-export signal (Ma et al., 2011), also supports interaction with the Golgin-97 grip domain. Thus, Kir2.1 cytoplasmic C-terminus has the capacity to orchestrate Golgi-97 binding, independently of TGN-export.

To test if the interaction is direct, His-tagged Golgin GRIP domains of Golgin-97 and Golgin-245 were produced in *E. coli*, purified and tested for binding in GST-affinity chromatography studies with purified GST-fusion proteins of the Kir2.1 C-terminal domain (WT and Δ314–315 mutant). As shown in **Figure 2F**, GST-Kir2.1C (WT and Δ314–315 mutant), but not GST alone, pulled down the Golgin-97 GRIP domain. The interaction appeared to be highly specific because the Golgin-245 grip domain was unable to interact with GST-Kir2.1CTCD; no detectable binding was observed even after prolonged exposure.

**FIGURE 3:**
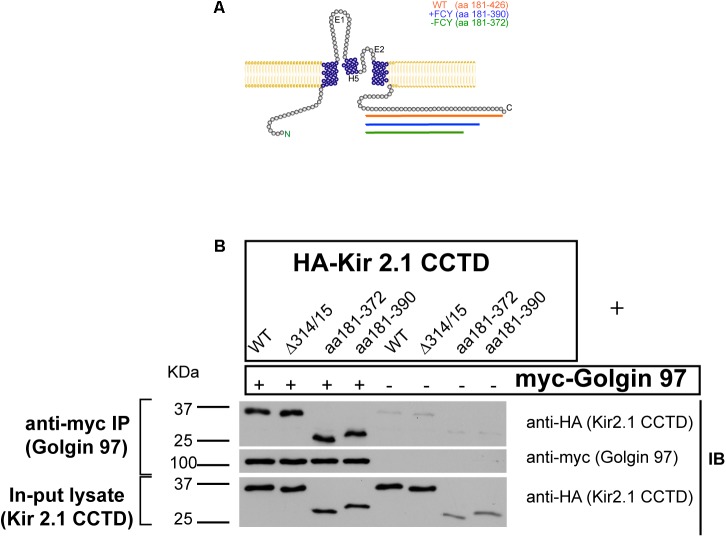
The Kir2.1 C-terminal cytoplasmic domain (CTCD) Interacts with Golgi-97 independently of ER/Golgi export and PDZ-binding signals. **(A)** Topological structure of Kir2.1, depicting C-terminal deletion mutants. **(B)** The HA-tagged Kir2.1 channel CTCD or deletion mutants, including Golgi-export Δ314/15 (aa181–426), ER export -FCY (aa181–372) or -PDZ (181–390), were co-transfected with myc-tagged Golgin-97, and cell lysates were subjected to immunoprecipitation anti-myc and then immunoblotted with anti-HA or anti-myc antibodies.

We wondered if Golgi-97 might interact with the “FCY” ER-export signal (Ma et al., 2001) or the PDZ binding site (Leonoudakis et al., 2004a) at the extreme C-terminus of Kir2.1. To test this, we transfected COS cells with myc-tagged Golgin-97 and HA-tagged fragments of the Kir2.1 C-terminus (**Figure 3A**), and performed anti-myc immunoprecipitation, anti-HA immunoblots (**Figure 3B**). CTCD fragments, included the full length polypeptide (aa 181–426); a CTCD deletion mutant, lacking the PDZ binding motif (aa 181–390); and another CTCD domain, lacking the FCY ER export signal (181–337). As shown in **Figure 3**, each of the CTCD fragments co-immunoprecipitated with Golgin-97. Because Golgin-97 interacts with Kir2.1, independently of the ER-export signal it unlikely that Golgin-97 controls ER- to-Golgi transport of Kir2.1.

### Residues in GRIP Domain Essential for the Interaction With Kir2.1

**FIGURE 4:**
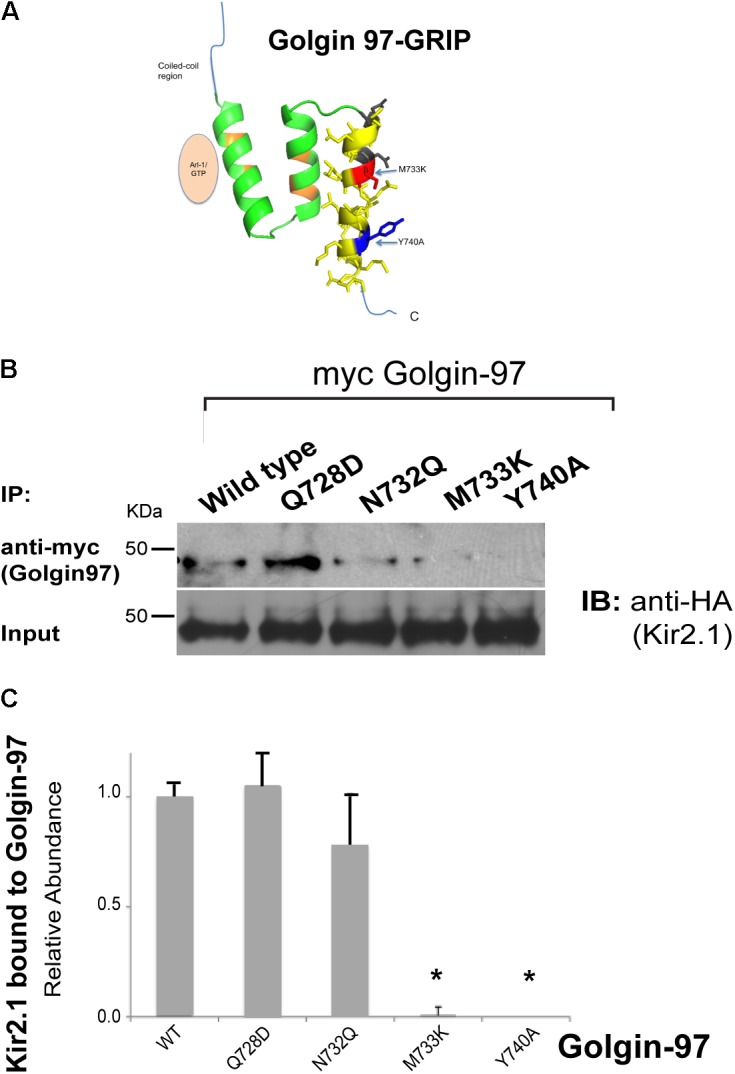
Mapping the residues in GRIP domain involved in Kir2.1 binding. **(A)** Model of the Golgin-97 GRIP domain. First helix directly interacts with Arl1, while the third helix contains residues (M733, Y740) that are necessary for binding to Kir2.1. **(B)** The HA-tagged Kir2.1 channel was co-transfected with myc-tagged Golgin-97 (WT or GRIP domain mutants), and cell lysates were subjected to immunoprecipitation with anti-myc and then immunoblotted with anti-HA to detect Kir2.1 bound. **(C)** Summary Data, Relative to Average WT Kir2.1 immunoprecipitated with wild type (WT) Golgin-97 GRIP. ^∗^*P* < 0.05.

We hypothesized that Kir2.1 might interact with the Golgin-97 GRIP domain at a site that is distinct from the Arl1-binding domain, and structural homology modeling with Golgin-245 (**Figure 4A**), suggested that the third helix as the most likely channel interaction site. Sequence analysis identified several residues in the third helix of the Golgi-97 GRIP domain that are different from the GRIP domain of Golgin-245. We replaced these residues with the corresponding amino acids found Golgin-245. As evaluated by co-immunoprecipitation, M733K and Y740A point mutations abrogated the binding of the GRIP domain to Kir2.1 (**Figures 4B,C**). Thus, M733 and Y740 are necessary for the interaction, presumably by acting as a direct docking site for Kir2.1.

### Interaction of Golgin-97 and Kir2.1 Occurs at the Golgi

**FIGURE 5:**
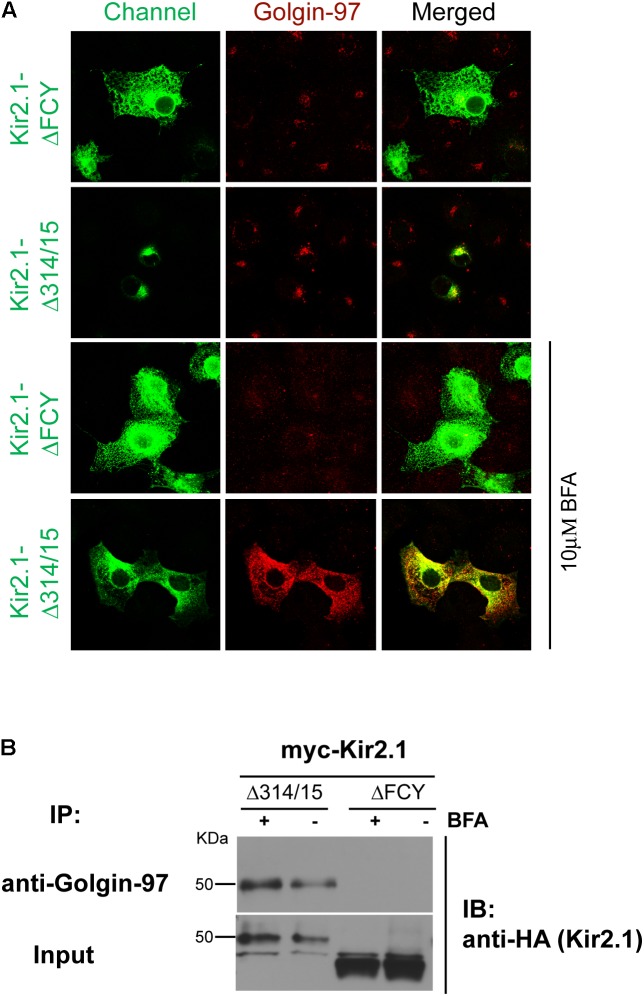
Golgin-97 Interacts with Kir2.1 in the Golgi. **(A)** Confocal images of Kir2.1 (TGN-export mutant (Δ314–315) compared to ER-export mutant ΔFCY) in COS-cells (green) and Golgi-97 (red) before (top) and after 10 min of brefeldin A treatment. **(B)** Anti-Golgin-97 immunoprecipitation (IP) followed by anti-HA immunoblotting (IB) from COS cells transfected with the HA-Kir2.1 mutants and treated with vehicle (–) or Brefeldin (+).

We next determined whether the Golgin-97-Kir2.1 interaction is initiated in the Golgi. For these studies, COS7 cells were transfected with two different Kir2.1 trafficking mutants, the ER-export deficient, ΔFCY, (Ma et al., 2001) or the Golgi-export deficient, Δ314–315 (Ma et al., 2011), and the channels were co-localized (**Figure 5A**) or co-immunoprecipitated (**Figure 5B**) with Golgi 97, before and after treatment with brefeldin A (Ward et al., 2001). As shown in **Figure 5**, the ΔFCY mutant channel largely resided in subcellular compartment, typical of the endoplasmic reticulum as shown previously (Ma et al., 2001), and did not appreciably co-localize (**Figure 5A**) or co-immunoprecipitate with Golgin-97 (**Figure 5B**), regardless of BFA treatment. By contrast, a large fraction of the Golgi-export mutant, Kir2.1 Δ314–315, interacted with and co-localized with Golgin-97 in a perinuclear compartment, typical of the Golgi as shown previously (Ma et al., 2011). Treatment with BFA stimulated the absorption of Kir2.1 Δ314–315 and Golgi-97 to the ER, where the two proteins remained co-localized (**Figure 5A**), and co-immunoprecipitated (**Figure 5B**). These observations are consistent with the idea that Golgin-97 channel binds to Golgin-97 in the *cis*- or medial Golgi en route to the TGN, and the proteins remain tightly associated even after BFA treatment.

### Knockdown of Golgin-97 Inhibits the Cell Surface Delivery of Kir2.1

**FIGURE 6:**
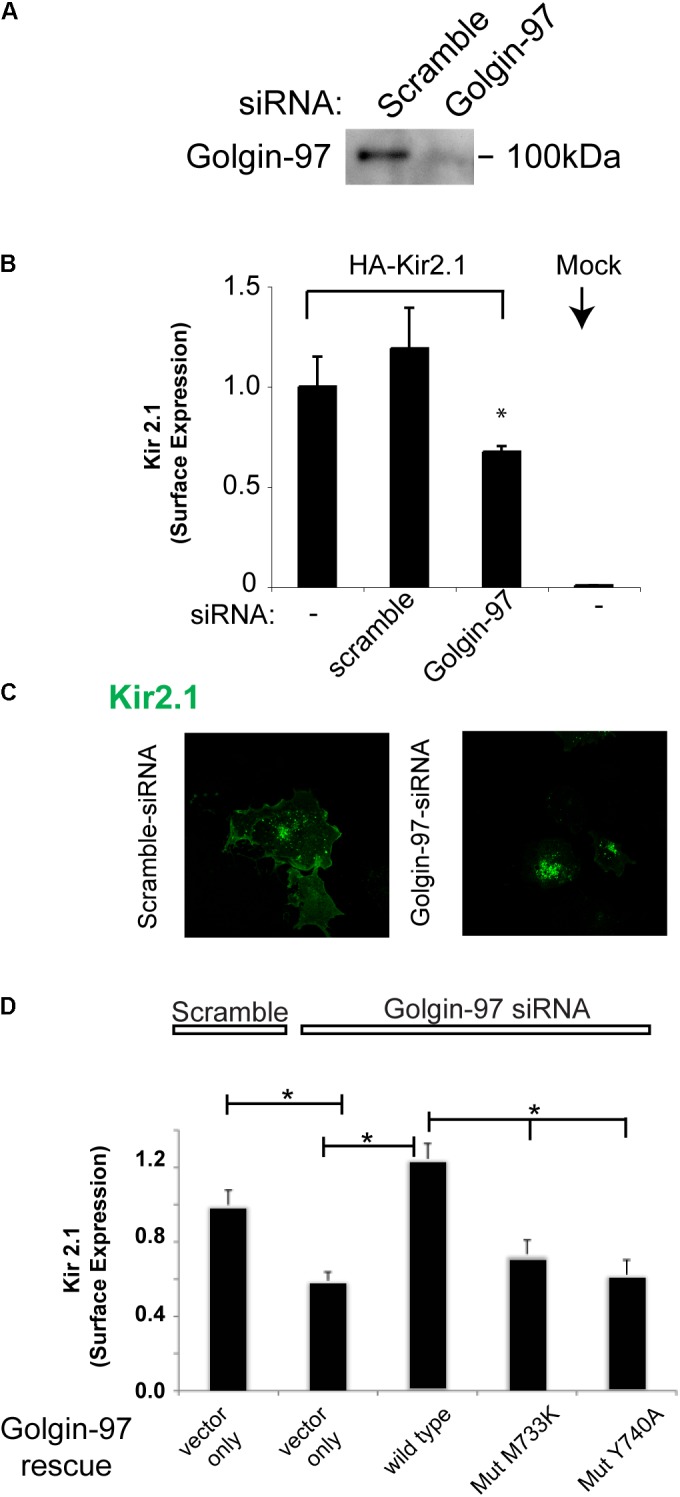
Golgin-97 is required for efficient delivery of Kir2.1 to the cell surface membrane from the Golgi. **(A)** Immunoblot of Golgin-97 from COS cells transfected with scramble or Golgi-97 siRNA. **(B)** Surface Expression of external HA-epitope tagged Kir2.1 in COS cells co-transfected with scramble or Golgi-97 siRNA or nothing. Mock are cells transfected with vector alone. **(C)** Representative confocal microscopy of Kir2.1 in COS cells transfected with scrample, or Golgin-97 siRNA. **(D)** Rescue Studies, Surface Expression of external HA-epitope tagged Kir2.1 in COS cells co-transfected with scramble or Golgi-97 siRNA and Golgin-97 rescue cDNA (WT vs mutant), ^∗^*P* < 0.05.

To evaluate the functional consequence of the interaction between Kir2.1 and Golgin-97, we assessed the impact of knocking down Golgi-97 on the subcellular localization and expression of Kir2.1 at the plasmalemma. Significant reduction in Golgin-97 protein abundance was achieved upon transfection with Golgin-97 specific siRNA, compared to the scramble probe (**Figure 6A**) in COS cells. As measured by surface anti-HA antibody binding and analytical luminometry, Golgin-97 depletion significantly reduced Kir2.1 expression on the surface membrane (**Figure 6B**). Immunocytochemistry and confocal microscopy revealed that Kir2.1 largely accumulated in the Golgi following Golgin-97 knockdown, while the scramble siRNA probe had no effect on the Kir2.1 distribution at the cell surface membrane (**Figure 6C**). We conclude from these studies that Golgin-97 is necessary for Kir2.1 export from the Golgi.

To test whether Golgin-97 drives Kir2.1 channel trafficking in a manner that is dependent on channel interaction, we performed rescue experiments with siRNA-resistant Golgi-97 constructs, and compared the rescue response of WT Golgi-97 to binding deficient (M733K and Y740A) mutants (**Figure 6D**). For these studies, COS-7 cells were co-transfected with the Golgin-97 constructs, Golgin-97 siRNA and the external epitope tagged Kir2.1. As measured by surface anti-HA antibody binding, expression of wild type Golgi-97, but not the binding deficient mutants, rescued the trafficking of Kir2.1 to the surface membrane in Golgin-97 knockdown cells. Thus, trafficking of Kir2.1 to the cell surface is dependent on Kir2.1 interaction with Golgin-97.

## Discussion

This study reveals a previously unappreciated trafficking process that directs potassium channels in the Golgi for delivery to the plasma membrane. It has been generally believed that secretory proteins are sorted in Golgi from resident Golgi proteins and proteins traveling to the ER by virtue of differences in transmembrane spans and lipid partitioning (Patterson et al., 2008). Similar processes have recently have been shown to segregate endolysosomal proteins from plasma membrane-destined proteins in the *cis*-Golgi for transport to distinct exit sites at the TGN (Chen et al., 2017). Our data suggest signal-dependent protein–protein interactions can also drive Golgi sorting processes. Indeed, we identified Golgin-97 as an interacting partner of Kir2.1, and found Gogin-97 is essential for Golgi-trafficking and delivery of Kir2.1 to the cell surface membrane. Because the interaction is initiated in the Golgi, we propose Golgin-97 marks the channel for inclusion in specialized exit sites at the TGN, where the Kir2.1 and related channels are incorporated into clathrin-coated vesicles through AP1 clathrin adaptor interactions (Ma et al., 2011).

The discovery of Golgin-97-dependent Kir2.1 trafficking extends our understanding of the Golgin molecules. Golgin-97 and Golgin p230 belong to a subset of the Golgin family members that interact with TGN membranes (Munro, 2011). Although both Golgins depend on Arl1 for TGN localization (Lu and Hong, 2003; Panic et al., 2003; Derby et al., 2004; Shin et al., 2005), they associate with distinct sets of vesicular carriers (Wong and Munro, 2014) and appear to orchestrate different trafficking processes. For instance, Golgin-p230 is required for TNF secretion (Lieu et al., 2008), whereas Golgi-97 is required for controlling exit of E-cadherin from the TGN (Lock et al., 2005). Golgin specificity is achieved, in part, by differences in vesicle-binding sequences found in the coiled-coil domains (Wong and Munro, 2014; Gillingham and Munro, 2016). In fact, the coiled-coil domain of Golgin-97 contains a specialized binding site for adaptor molecules that associate with donor vesicles (Wong and Munro, 2014). Our observations reveal an additional mechanism. By specifically interacting with Kir2.1 through the GRIP domain, Golgin-97 specifies trafficking at the acceptor membrane. It will be important to learn if other Golgin-97 dependent forward trafficking processes, such as Golgi export of E-cadherin (Lock et al., 2005), are also orchestrated by direct binding interactions at the acceptor membrane.

Although Golgin-97 is required for efficient anterograde trafficking of Kir2.1 and E-cadherin from the TGN (Lock et al., 2005), it also mediates the retrograde transport of endosomes to the TGN (Shin et al., 2017; Wong et al., 2017). We speculate that these seemingly opposite functions are interrelated. Similar to other Golgins, which facilitate endosome-to-Golgi transport to maintain Golgi structure (Derby et al., 2007), Golgin-97 may facilitate retrograde transport of specific endosomes to recycle trafficking machineries and, thereby maintain TGN sub-domains that are competent for the export of Kir2.1 and E-cadherin. Because Kir2.1 (Ma et al., 2011) and E-cadherin (Hase et al., 2013) share a common AP1 clathrin-adaptor dependent Golgi export mechanism, we speculate Golgin-97 mediated endosome-to-TGN transport allows recycling of the appropriate compliment of proteins that are required to nucleate AP1-dependent export carriers, reminiscent of the way that GCC185 recycles AP1 (Derby et al., 2007; Brown et al., 2011).

We found binding site for Golgin-97 is embedded within the large cytoplasmic region of the Kir2.1 C-terminus. Significantly, the binding site is distinct from other known trafficking motifs in the channel, including the Golgi-export signal (Ma et al., 2011), the ER-export signal (Ma et al., 2001), and the PDZ binding motif (Leonoudakis et al., 2004a). The region harbors several residues that are mutated in Andersen-Tawil syndrome (Plaster et al., 2001), and that are known to be required for efficient surface expression (Bendahhou et al., 2003). It will be interesting to learn if these residues form the interaction site for Golgin-97.

There are several limitations of this study that deserve discussion. First, this study focused on membrane trafficking of Kir2.1 in a heterologous expression system. It presently remains to be determined if Golgin-97 controls Kir2.1 in a similar way in tissues where the channel is endogenously expressed, including the heart, skeletal muscle, smooth muscle, and endothelium. Studying endogenous Kir 2.1 trafficking in native tissue is currently not practical, however. Native expression levels are much lower than in experimental expression systems and antibodies are lacking sufficient efficacy to detect the native channel protein with sufficient specificity. Development of new tools will be required to test the Golgi-97 dependent trafficking mechanism in native tissues, such as by engineering an epitope tag on the channel gene, and then studying endogenous tagged-channel in cells isolated from the genetically modified model. Second, these studies were designed to study membrane trafficking of Kir2.1, not necessarily channel activity. It will be interesting for future studies to determine whether Golgi-97 as any direct effects on channel activity.

In summary, Golgin-97 directly interacts with Kir2.1 to control anterograde transport at the Golgi. Signal-dependent interactions with Golgi tethering proteins provide a mechanism to target interacting proteins to specialized TGN sub-domains to facilitate trafficking to appropriate subcellular membrane domains.

## Author Contributions

TT and DM designed and executed the studies, analyzed the results and prepared the figures. BK executed the studies involving cDNA cloning and mutagenesis. PW designed the studies and prepared the manuscript.

## Conflict of Interest Statement

The authors declare that the research was conducted in the absence of any commercial or financial relationships that could be construed as a potential conflict of interest.
